# Multi-Path Optimization for Efficient Production of 2′-Fucosyllactose in an Engineered *Escherichia coli* C41 (DE3) Derivative

**DOI:** 10.3389/fbioe.2020.611900

**Published:** 2020-12-03

**Authors:** Zhijian Ni, Zhongkui Li, Jinyong Wu, Yuanfei Ge, Yingxue Liao, Lixia Yuan, Xiangsong Chen, Jianming Yao

**Affiliations:** ^1^Institute of Plasma Physics, Hefei Institutes of Physical Science, Chinese Academy of Sciences, Hefei, China; ^2^University of Science and Technology of China, Hefei, China; ^3^Wuhan Zhongke Optics Valley Green Biotechnology Co. Ltd., Wuhan, China

**Keywords:** *Escherichia coli*, fucosylation, GDP-D-mannose, GDP-L-fucose, 2′-fucosyllactose

## Abstract

2′-fucosyllactose (2′-FL), one of the simplest but most abundant oligosaccharides in human milk, has been demonstrated to have many positive benefits for the healthy development of newborns. However, the high-cost production and limited availability restrict its widespread use in infant nutrition and further research on its potential functions. In this study, on the basis of previous achievements, we developed a powerful cell factory by using a *lacZ*-mutant *Escherichia coli* C41 (DE3)ΔZ to ulteriorly increase 2′-FL production by feeding inexpensive glycerol. Initially, we co-expressed the genes for GDP-L-fucose biosynthesis and heterologous α-1,2-fucosyltransferase in C41(DE3)ΔZ through different plasmid-based expression combinations, functionally constructing a preferred route for 2′-FL biosynthesis. To further boost the carbon flux from GDP-L-fucose toward 2′-FL synthesis, deletion of chromosomal genes (*wcaJ, nudD*, and *nudK*) involved in the degradation of the precursors GDP-L-fucose and GDP-mannose were performed. Notably, the co-introduction of two heterologous positive regulators, RcsA and RcsB, was confirmed to be more conducive to GDP-L-fucose formation and thus 2′-FL production. Further a genomic integration of an individual copy of α-1,2-fucosyltransferase gene, as well as the preliminary optimization of fermentation conditions enabled the resulting engineered strain to achieve a high titer and yield. By collectively taking into account the intracellular lactose utilization, GDP-L-fucose availability, and fucosylation activity for 2′-FL production, ultimately a highest titer of 2′-FL in our optimized conditions reached 6.86 g/L with a yield of 0.92 mol/mol from lactose in the batch fermentation. Moreover, the feasibility of mass production was demonstrated in a 50-L fed-batch fermentation system in which a maximum titer of 66.80 g/L 2′-FL was achieved with a yield of 0.89 mol 2′-FL/mol lactose and a productivity of approximately 0.95 g/L/h 2′-FL. As a proof of concept, our preliminary 2′-FL production demonstrated a superior production performance, which will provide a promising candidate process for further industrial production.

## Introduction

Breast-feeding has long been considered the preferred option for infant nutrition, since human milk contains a large number of bioactive nutrients that are not easily available and indispensable (Bode, 2012). Therein, human milk oligosaccharides (HMOs) provide a wide range of biological functions for infant care, for instance, improvement of infant digestion and intestinal development, promotion of infant brain development, and immune maturation (Bode, 2012; Donovan and Comstock, 2016; Reverri et al., 2018). In particular, 2′-fucosyllactose (2′-FL), a trisaccharide composed of L-fucose and D-lactose, is one of foremost functional component with the highest content in HMOs (Castanys-Muñoz et al., 2013), and its curraceutical foods and pharmaceutical purposes has gained much interest (Jennewein Biotechnologie Gmb, 2011, 2014; Nestlé, 2019; Abbott Laboratories, 2020; Wyeth Nutrition, 2020). Importantly, 2′-FL has been officially approved for use as a novel food additive in infant formula to improve probiotic functions by USA Food and Drug Administration (FDA) and the European Food Safety Authority (EFSA) (Sprenger et al., 2017; Bych et al., 2019). However, as a naturally-occurring ingredient with a high content in human breast milk, 2′-FL is almost absent in cow's milk which is the most commonly used raw material for infant formula (Petschacher and Nidetzky, 2016). Hence, industrial 2′-FL production cost-effectively has attracted more and more attention, and has become the focus of competition in patent protection worldwide.

**Figure 1:**
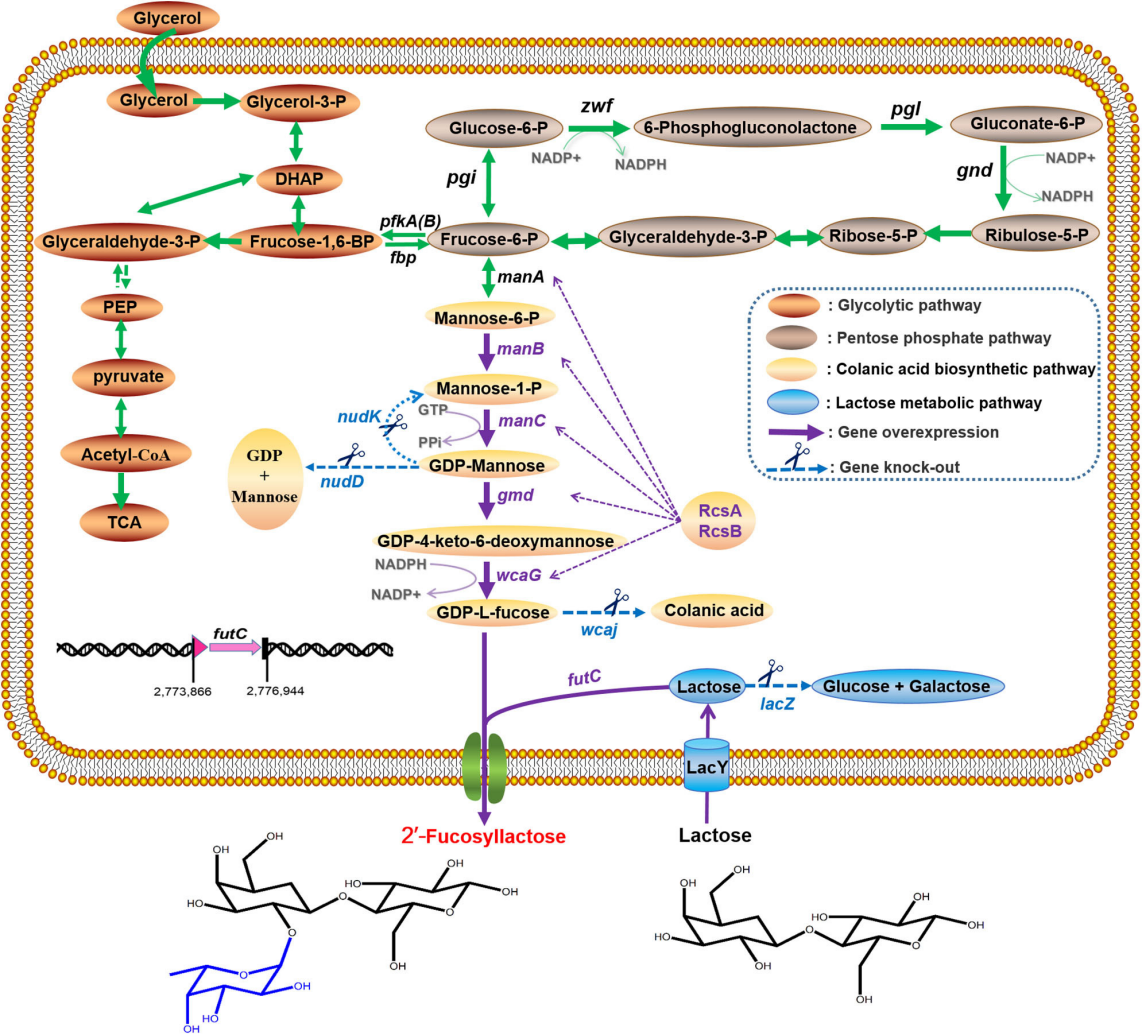
Schematic representation of 2′-FL production by metabolically engineered *E. coli* in this study. The intracellular enzymes are abbreviated as follows: Zwf, glucose-6-phosphate dehydrogenase; Pgl, 6-phosphogluconolactonase; Gnd, 6-phosphogluconate dehydrogenase; Pgi, phosphoglucose isomerase; PfkA(B), 6-phosphofructokinase-1(2); ManA, mannose 6-phosphate isomerase; ManB, phosphomannomutase; ManC, α-D-mannose 1-phosphate guanylyltransferase; Gmd, GDP-mannose 6-dehydrogenase; WcaG, GDP-L-fucose synthase; WcaJ, UDP-glucose: undecaprenyl-phosphate glucose-1-phosphate transferase; NudK, GDP-mannose hydrolase; NudD, GDP-mannose mannosyl hydrolase; RcsA, positive transcriptional regulator A; RcsB, positive transcriptional regulator B; LacZ, β-galactosidase; LacY, lactose permease; FutC, α-1,2-fucosyltransferase.

Over the years, the area of HMOs research has seen a tremendous development, and several approaches to produce 2′-FL have been reviewed in publications (Han et al., 2012; Sprenger et al., 2017; Bych et al., 2019), including chemical synthesis, enzymatic conversion, and microbial fermentation. Currently, biosynthesis of 2′-FL in engineered microbial systems is considered as the mainstream method because it allows large-scale production with simple processes (Han et al., 2012; Becker and Wittmann, 2016; Bych et al., 2019). For whole-cell synthesis of 2′-FL, the concurrent presence of three components must be continuously requisite, namely donor-substrate GDP-L-fucose, acceptor lactose and α-1,2-fucosyltransferase (FT). Among these, lactose as an inexpensive substrate can generally be assimilated and degraded by wild-type producer itself, resulting in a decrease in lactose utilization. For this reason, certain engineering strategies (Lee et al., 2012a; Chin et al., 2015; Huang et al., 2017), such as eliminating β-galactosidase activity while retaining or enhancing lactose uptake, have achieved positive performance to allow formation of 2′-FL. GDP-L-fucose is a key precursor for the synthesis of fucosylated HMOs, and it can be synthesized through two pathways (Sprenger et al., 2017). One is the *de novo* pathway naturally present in *E. coli* originating from colanic acid biosynthesis (Stevenson et al., 1996). Starting from fructose 6-phosphate in the central metabolism, it is converted into GDP-L-fucose via five enzymatic steps (ManA, ManB, ManC, Gmd, WcaG) (Figure 1). The other is the *salvage* pathway originally derived from eukaryotic cells, which requires an expensive L-fucose as a substrate and the catalytic enzyme Fkp from *Bacteroides fragilis* (*B. fragilis*) to produce GDP-L-fucose (Chin et al., 2016). In brief, the intracellular availability of GDP-L-fucose is of great importance for 2′-FL production, and the *de novo* pathway are usually used as the preferred route for GDP-L-fucose synthesis. In addition, it has been proved that the inactivation of *lon* and/or *wcaJ* genes involved in the biosynthesis of colonic acid, and/or the overexpression of the positive regulator gene *rcsA*, can increase the intracellular supply of GDP-L-fucose (Drouillard et al., 2006; Huang et al., 2017), but the shortage of certain intermediates (e.g., GDP-mannose) in this pathway may still weaken the carbon flux toward GDP-L-fucose formation, indicating that this will become an innovative point for our work. More importantly, to generate 2′-FL from lactose and GDP-L-fucose present, a highly active α-1,2-FT sufficient in the cytoplasm is always necessary to facilitate the fucosylation reaction. Previous studies have identified a variety of various FT candidates, some of which, such as the *futC* gene from *Helicobacter pylori* (*H. pylori*) or *wcfB* gene from *B. fragilis* for an α-1,2-FT, provided the clear opportunities for efficient 2′-FL synthesis *in vivo* (Chin et al., 2017; Huang et al., 2017; Deng et al., 2019).

Extensive efforts have been reported for 2′-FL production with engineered *E. coli* cells (Lee et al., 2012a; Baumgärtner et al., 2013; Chin et al., 2015, 2016, 2017; Huang et al., 2017; Supplementary Table 2), but the laboratory-scale 2′-FL was produced at below 30 g/L, which is obviously not sufficient to support the cost-effective production. Just recently, the group of Jung et al. (2019) based their approach on the *salvage* pathway achieved a high titer of 47.0 g/L 2′-FL with a yield of 0.52 mole 2′-FL/mole fucose in their fed-batch fermentation by eliminating the degradation of lactose and L-fucose (Jung et al., 2019). However, the limited source and high price of L-fucose increase the costs of 2′-FL production, thus hindering its application in large-scale production. *E. coli* BL21 (DE3) is a powerful and widely used host for the mass-production of many bioproducts (Kim et al., 2018), and 2′-FL production using engineered BL21 (DE3) has been released with a good yield in the batch fermentation (Huang et al., 2017), but closely large-scale production performance has not been confirmed. Another point worth mentioning is that in practice, overexpression of certain proteins in the BL21 (DE3) with a strong transcription of T7 RNA polymerase may induce a metabolic burdens, such as growth inhibition, cell lysis, or even death (Bhattacharya and Dubey, 1995; Li and Rinas, 2020), which is not conducive to the industrial production of 2′-FL. *E. coli* C41 (DE3), a BL21 (DE3) mutant with a lower transcription rate of T7 RNA polymerase, can effectively relieve the toxic effect caused by overexpression of recombinant proteins (Dumon-Seignovert et al., 2004; Kwon et al., 2015). In this study, on the basis of a large body of previous achievements reported, we developed an engineered cell factory by using a *lacZ*-mutant *E. coli* C41 (DE3)ΔZ to ulteriorly efficiently produce 2′-FL. As illustrated in Figure 1, we first screened a reliable and efficient route for 2′-FL generation in the host cells from different plasmid-based expression combinations for co-expressing the genes for GDP-L-fucose biosynthesis from the *de novo* pathway and heterologous α-1,2-FT. Then the chromosomal genes, *wcaJ, nudD*, and *nudK*, were completely deleted to enhance 2′-FL production. Moreover, a pair of heterologous positive regulators, RcsA and RcsB, were also attempted to promote GDP-L-fucose formation and thus 2′-FL production. Further a genomic integration of an individual copy of α-1,2-FT gene, as well as the preliminary optimization of fermentation conditions enabled the resulting engineered strain to achieve a high titer and yield. By collectively taking into account the intracellular lactose utilization, GDP-L-fucose availability, and fucosylation activity for 2′-FL production, finally the feasibility of large-scale production was demonstrated in a modified 50-L fed-batch fermentation system.

## Materials and Methods

### Bacterial Strains and Plasmids

All strains and plasmids used in this study are summarized in Table 1. *E. coli* DH5α was used as a host strain for cloning and construction of plasmids. *E. coli* K12 MG1655 provided the genomic template for obtaining genes of interest. *E. coli* C41 (DE3)ΔZ deficient in β-galactosidase was previously constructed by Dr. Jinyong Wu in our laboratory, and used as the host strains for 2′-FL production in this study. Vectors pACYCDuet-1, pCDFDuet-1, pRSFDuet-1, and pETDuet-1 (Novagen) were used for cloning and subcloning. pCas (Addgene, #62225) and pTargetF (Addgene, #62226) were purchased from Addgene for CRISPR/Cas9-meditated genome editing. All primers used in this study are listed in Supplementary Table 1, and were synthesized at Sangon Biotech (Shanghai, China).

**Table 1:** Strains and plasmids used in this study.

**Names**	**Genotypes**	**References**
**Strains**		
*E. coli* DH5α	F^−^*endA1 glnV44 thi-1 recA1 relA1 gyrA96 deoR nupG* Φ80d*lacZ*ΔM15Δ(*lacZYA-argF*)*U169 hsdR17*(rK^−^mK^+^)λ^−^	Invitrogen
*E. coli* K12 MG1655	F^−^λ^−^*ilvG-rfb-50 rph-1*	Invitrogen
*E. coli* C41 (DE3)	F^−^ ompT hsdSB (rB- mB-) gal dcm (DE3)	Thermo Fisher Scientific
C41 (DE3)ΔZ	*E. coli* C41 (DE3): Δ*lacZ*	Lab stock
C41 (DE3)ΔZW	*E. coli* C41 (DE3): Δ*lacZ*Δ*wcaJ*	This study
C41 (DE3)ΔZWD	*E. coli* C41 (DE3): Δ*lacZ*Δ*wcaJ*Δ*nudD*	This study
C41 (DE3)ΔZWK	*E. coli* C41 (DE3): Δ*lacZ*Δ*wcaJ*Δ*nudK*	This study
C41 (DE3)ΔZWD-F	*E. coli* C41 (DE3): Δ*lacZ*Δ*wcaJ*Δ*nudD*Δ*fucIK*::P_T7_-*futC*	This study
C41ΔZ/pA	C41 (DE3)ΔZ harboring plasmids pAC-CBGW and pET-*futC*	This study
C41ΔZ/pC	C41 (DE3)ΔZ harboring plasmids pCD-CBGW and pET-*futC*	This study
C41ΔZ/pR	C41 (DE3)ΔZ harboring plasmids pRS-CBGW and pET-*futC*	This study
C41ΔZW/pR	C41 (DE3)ΔZW harboring plasmids pRS-CBGW and pET-*futC*	This study
C41ΔZWD/pR	C41 (DE3)ΔZWD harboring plasmids pRS-CBGW and pET-*futC*	This study
C41ΔZWK/pR	C41 (DE3)ΔZWK harboring plasmids pRS-CBGW and pET-*futC*	This study
C41ΔZWD/pRA	C41 (DE3)ΔZWD harboring plasmids pRS-CBGW and pET-*rcsA-futC*	This study
C41ΔZWD/pRAB	C41 (DE3)ΔZWD harboring plasmids pRS-CBGW and pET-*rcsAB-futC*	This study
C41ΔZWD-F/pRAB	C41 (DE3)ΔZWD-F harboring plasmids pRS-CBGW and pET-*rcsAB-futC*	This study
**Plasmids**		
pACYCDuet-1	Two T7 promoters, p15A replicon, Cm^R^, low copy number, 10 ~ 12 copies/cell	Novagen
pCDFDuet-1	Two T7 promoters, CloDF13 replicon, Sm^R^, medium copy number, 20 ~ 40 copies/cell	Novagen
pRSFDuet-1	Two T7 promoters, RSF1030 replicon, Kan^R^, high copy number, ~100 copies/cell	Novagen
pETDuet-1	Two T7 promoters, pBR322 replicon, Amp^R^, medium copy number, ~40 copies/cell	Novagen
pAC-CBGW	pACYCDuet-1+ *manC-manB-*T7 terminator (NcoI/NotI) + *gmd-wcaG* (NdeI/AvrII), Cm^R^	This study
pCD-CBGW	pCDFDuet-1 + *manC-manB*- T7 terminator (NcoI/NotI) + *gmd-wcaG* (NdeI/AvrII), Sm^R^	This study
pRS-CBGW	pRSFDuet-1 + *manC-manB-* T7 terminator (NcoI/NotI) + *gmd-wcaG* (NdeI/AvrII), Kan^R^	This study
pET-*futC*	pETDuet-1 + *futC* (NdeI/AvrII), Amp^R^	This study
pET-*rcsA*-*futC*	pET-*futC* + *rcsA*(NcoI/HindIII), Amp^R^	This study
pET-*rcsAB*-*futC*	pET-*rcsA*-futC *+ rcsB*(HindIII/NotI), Amp^R^	This study
pKD-*futC*-FKF	pKD4 + P_T7_-*futC2*-Terminator (NdeI/NotI), Kan^R^	This study
pKD46	Expressed λ-red recombinase (gam, bet, exo), *repA101*(Ts) *bla araC P_*araB*_-Red*, Amp^R^	Datsenko and Wanner, 2000
pKD4	*FRT aph FRT PS1 PS2 oriR6K*, Kan^R^	Datsenko and Wanner, 2000
pCP20	*cI857 λP_*R*_ flp pSC101 oriTS*, Amp^R^ Cm^R^	Cherepanov and Wackernagel, 1995
pCas	*repA101*(Ts) *kan P_*cas*_-cas9 P_*araB*_-Red lacI^*q*^ P_*trc*_*-sgRNA*-pMB1*, Kan^R^	Addgene (#62225)
pTargetF	*pMB1 aadA* sgRNA, Amp^R^, Constitutive expression of sgRNA without donor editing template DNA	Addgene (#62226)
pTargetF-*wcaj*	*pMB1 aadA* sgRNA-*wcaJ*, AmpR, The specific N20 sequence target to *wcaJ* gene	This study
pTargetF-*nudD*	*pMB1 aadA* sgRNA-*nudD*, Amp^R^, The specific N20 sequence target to *nudD* gene	This study
pTargetF-*nudK*	pMB1 aadA sgRNA-*nudK*, Amp^R^, The specific N20 sequence target to *nudK* gene	This study

To strengthen the GDP-L-fucose biosynthesis in host cells, plasmids, pAC-CBGW, pCD-CBGW, pRS-CBGW, with different copy numbers for overexpression of the genes encoding GDP-L-fucose biosynthetic enzymes (ManB, ManC, Gmd, and WcaG) were constructed individually with the parent vectors pACYCDuet-1, pCDFDuet-1, and pRSFDuet-1 using ClonExpress^®^ II One Step Cloning Kit (Vazyme, Nanjing, China) according to the manufacturer's instructions. The α-1,2-FT gene, *futC*, from *H. pylori* was codon-optimized and commercially synthesized by Sangon Biotech (Shanghai, China), and then ligated to pETDuet-1 digested with NdeI/AvrII to form pET-*futC*. Similarly, the genes, *rcsA* and *rcsB*, from the genomic DNA of *E. coli* MG1655 were cloned successively into pET-*futC*, resulting in pET-*rcsA-futC* and pET-*rcsAB-futC*. In addition, an intact expression cassette of *futC* gene driven by a T7 promoter was installed from plasmid pET-*futC* into pKD4 digested with NdeI/NotI to generate an integrative vector pKD-FT-FKF containing FRT-kan-FRT cassette. To knock out genes from the *E. coli* chromosome, several pTargetF plasmids carrying 20 bp target sequence (N20) unique for each gene locus of interest were routinely constructed by reverse PCR from native pTargetF (Addgene, #62226) to express the targeting guide RNA sequences. Each N20 sequence was synthesized at the 5′-ends of primers as shown in Supplementary Table 1. All plasmids constructed are depicted in detail in Supplementary Figure 1, and confirmed by DNA sequencing.

### Genomic Manipulations

Deletion of chromosomal genes (*wcaJ, nudD*, and *nudK*) from *E. coli* C41 (DE3)ΔZ were carried out using the CRISPR-Cas9 technology as reported previously (Jiang et al., 2015). Specifically, a single colony of *E. coli* harboring pCas plasmid was inoculated into LB containing 50 ug/ml kanamycin and induced with 20 mM arabinose for expression of λ-red recombinase prior to preparing electro-competent cells. Approximately 500 bp homologous arms were separately amplified from the upstream and downstream of the target gene loci, and then fused together by overlap PCR to prepare donor dsDNA fragments for the genome editing templates. The sequences of donor dsDNA fragments are provided in Supplementary Table 1. For each electrotransformation, 100 ng of specific pTargetF and 400 ng of dsDNA fragment were well-mixed with 50 μl aliquots of competent cells containing Cas9 protein and λ-red recombinase, and then electroporated in a 1-mm cuvette (Bio-Rad) at 1.80 kV using Bio-Rad MicroPulser (Shanghai, China). Instantly, the shocked cells were suspended in 1 ml of ice-cold fresh medium and recovered for 2 h at 30°C. As a result, the mutants were identified from an agar plate by colony PCR and DNA sequencing. Eventually, plasmids pTargetF and pCas were removed as described by Jiang et al. (2015).

For the chromosomal integration of α-1,2-FT gene (*futC*), an individual expression cassette of *futC* containing the kanamycin-resistance gene flanked by FRT sites was amplified by PCR from the pre-constructed plasmid pKD-FT-FKF (Supplementary Figure 1G). And the purified PCR product was intergrated into the *fucIK* gene locus of *E. coli* strain harboring pKD46 using the λ-Red recombinase method as described previously (Datsenko and Wanner, 2000; Baumgärtner et al., 2013). The insertion of foreign DNA sequence was confirmed by the colony PCR and DNA sequencing. For the correct mutant, the antibiotic resistance cassette was eliminated by pCP20 expressing FLP recombinase.

### Culture Conditions and Bacterial Fermentation

In all cases unless otherwise noted, *E. coli* strains were cultured in liquid LB medium (containing 10 g/L tryptone, 5 g/L yeast extract and 10 g/L NaCl) at 37°C with shaking at 220 rpm for cell growth. Moreover, as required, appropriate antibiotics (34 μg/mL chloramphenicol, 50 μg/mL kanamycin, 100 μg/mL ampicillin, and 50 μg/mL spectinomycin) were added into the medium at 30°C or 37°C for single-clone selection, plasmid preparation, and maintenance.

To detect 2′-FL production in different recombinant strains, batch fermentations were done in 500-mL baffled shake flasks (Nalgene) containing 100 ml of the defined medium I with 30 g/L glycerol as a carbon source. Specifically speaking, the inocula were pre-cultured at 37°C and 220 rpm in the shake flasks. When the optical density at 600 nm (OD600) reached 0.8, IPTG was added at the final concentration of 0.2 mM for induction at 25°C and 250 rpm. After 2 h of additional cultivation, 10.0 g/L lactose as an acceptor substrate was injected for 2′-FL production. The defined medium I was prepared as described by Huang et al. (2017) with some modifications, namely 5.0 g/L yeast extract, 10.0 g/L tryptone, 17.1 g/L Na_2_HPO_4_·12H_2_O, 1.0 g/L (NH_4_)_2_HPO_4_, 3.0 g/L KH_2_PO_4_, 2.0 g/L NH_4_Cl, 2.0 g/L trisodium citrate, 1.4 g/L MgSO_4_·7H_2_O, 10.0 mg/L thiamine, and 1.0 mL/L trace metal solution. The trace metal solution composed of 25.0 g/L FeCl_3·_6H_2_O, 2.3 g/L CaCl_2_·2H_2_O, 2.6 g/L ZnCl_2_, 2.0 g/L CuSO_4_·5H_2_O, 2.5 g/L MnSO_4_·H_2_O, 2.6 g/L Na_2_MoO_4_·2H_2_O, and 0.7 g/L H_3_BO_3_, pH 6.9. Fed-batch fermentation was performed in a 50-L bioreactor (Bxbio, Shanghai, China) preloading 30-L of defined medium II containing 20 g/L glycerol as the initial carbon source. 1.5-L of pre-cultures were incubated in 1,000-ml shake flasks at 37°C and 220 rpm for 6 h, and then totally transferred into the bioreactor. During the pre-fermentation stage, the cultivation was performed at 37°C with an aeration rate of 2 vvm at 900 rpm. When the initial glycerol was completely exhausted, feeding solution containing 800 g/L glycerol and 30 g/L MgSO_4_·7H_2_O was fed into the bioreactor at a constant speed of 3.8 g/L/h, and the temperature was shifted to 25°C or 28°C. Simultaneously, IPTG was added to a final concentration of 0.2 or 0.3 mM for the induction of the T7 promoter-mediated gene expression. After additional cultivation for 2, 14, 26, and 38 h, same amount of lactose (roughly 22 g/L) was intermittently supplemented for four times to allow a continuous accumulation of 2′-FL. Throughout the entire cultivation, the pH of the fermentation broth was maintained at 6.8 by addition of 28% NH_4_OH using a pH-stat mode, and dissolved oxygen was controlled between 30 and 50% by automatically adjusting agitation speed. The defined medium II contained 8.0 g/L yeast extract, 12.0 g/L tryptone, 1.0 g/L citric acid, 13.2 g/L K_2_HPO_4_, 9.3 g/L KH_2_PO_4_, 4.0 g/L (NH_4_)_2_SO_4_, 1.4 g/L MgSO_4_·7H_2_O, 10 mg/L thiamine, and 1 mL trace metal solution.

### Analytical Methods

Cell growth was monitored by measuring OD600 using a UV5100-spectrophotometer (Metash, Shanghai, China), and biomass was expressed as dry cell weight (DCW) by a pre-determined conversion factor (0.372).

To determine the intracellular concentration of GDP-L-fucose, the cells harvested from 10 mL of the fermentation broth were washed with 10 mL of deionized water, then re-suspended in 1 mL of cell lysis buffer as described by Lee et al. (2009). The re-suspended cells were further disrupted using an ultrasonic processor (Shengxi, Shanghai, China) before being boiled at 100°C for 1 min. The supernatant was collected and analyzed using an HPLC system (LC-15C; Shimadzu, Kyoto, Japan) equipped with a C18 column and a UV detector (GL Sciences, Kyoto, Japan). The mobile phase consisted of solvent A (20 mM triethylamine acetate, pH 6.0) and solvent B (100% acetonitrile). The column equilibrated at 30°C was eluted at a flow rate of 0.6 mL/min with the following modified gradient program: 0~15 min, a linear gradient from 0 to 2% solvent B; 15~25 min, a linear gradient from 2 to 4% solvent B; 25 ~ 40 min, a linear gradient from 4 to 2% solvent B; and 40 ~ 45 min, a linear gradient from 2 to 1% solvent B. The peak response of GDP-L-fucose was detected at 254 nm. In addition, extracellular concentrations of glycerol, lactose, 2′-FL in the supernatants of the fermentation broth were measured by using an Agilent HPLC system (1,200 Series) equipped with a refractive index (RI) detector (Agilent) and an Aminex HPX-87H column (Bio-Rad). The column heated at 60°C was eluted with 5 mM H_2_SO_4_ solution as a mobile phase at a flow rate of 0.5 mL/min.

### Identification of 2′-FL Production by LC/MS Analysis

Prior to mass spectrometry analysis, the supernatants separated from the fermentation broth were purified and condensed by solid-phase extraction (SPE) with a porous graphitized carbon cartridge. The resulting samples was lyophilized, re-dissolved in 50% methanol and further analyzed by the LC/MS MALDI time-of-flight system (Waters Corp., Milford, MA, United States) according to the previous study reported by Yu et al. (2018). The scan range for MS consisted of a mass-to-charge ratio (m/z) of 100 ~ 600.

### β-Galactosidase Activity Assay

The β-galactosidase activity was determined by a basic colorimetric assay as described by Miller (1972). Cells were cultivated in 100 mL of the defined medium I with 30 g/L glycerol at 25°C and 250 rpm. After 6 h IPTG induction, cells were harvested by centrifugation at 4°C, then washed twice with ice-cold potassium phosphate buffer (20 mM, pH 7.2). The re-suspended cells were disrupted by an ultrasonic processor, and the supernatant was isolated and used for the enzymatic assays. After performing a Bradford assay (Harlow and Lane, 2006) to determine overall protein concentration in the supernatant, an aliquot of the supernatant is mixed with the substrate, O-nitrophenyl-β-D-galactopyranoside (ONPG), in a buffer containing sodium phosphate and magnesium chloride. When the yellow product becomes visible, OD420 are determined by a spectrophotometer. The assay was repeated independently in triplicate.

## Results

### 2′-FL Biosynthesis in the Recombinant *E. coli* C41 (DE3)ΔZ With Different Plasmid Combinations

**Figure 2:**
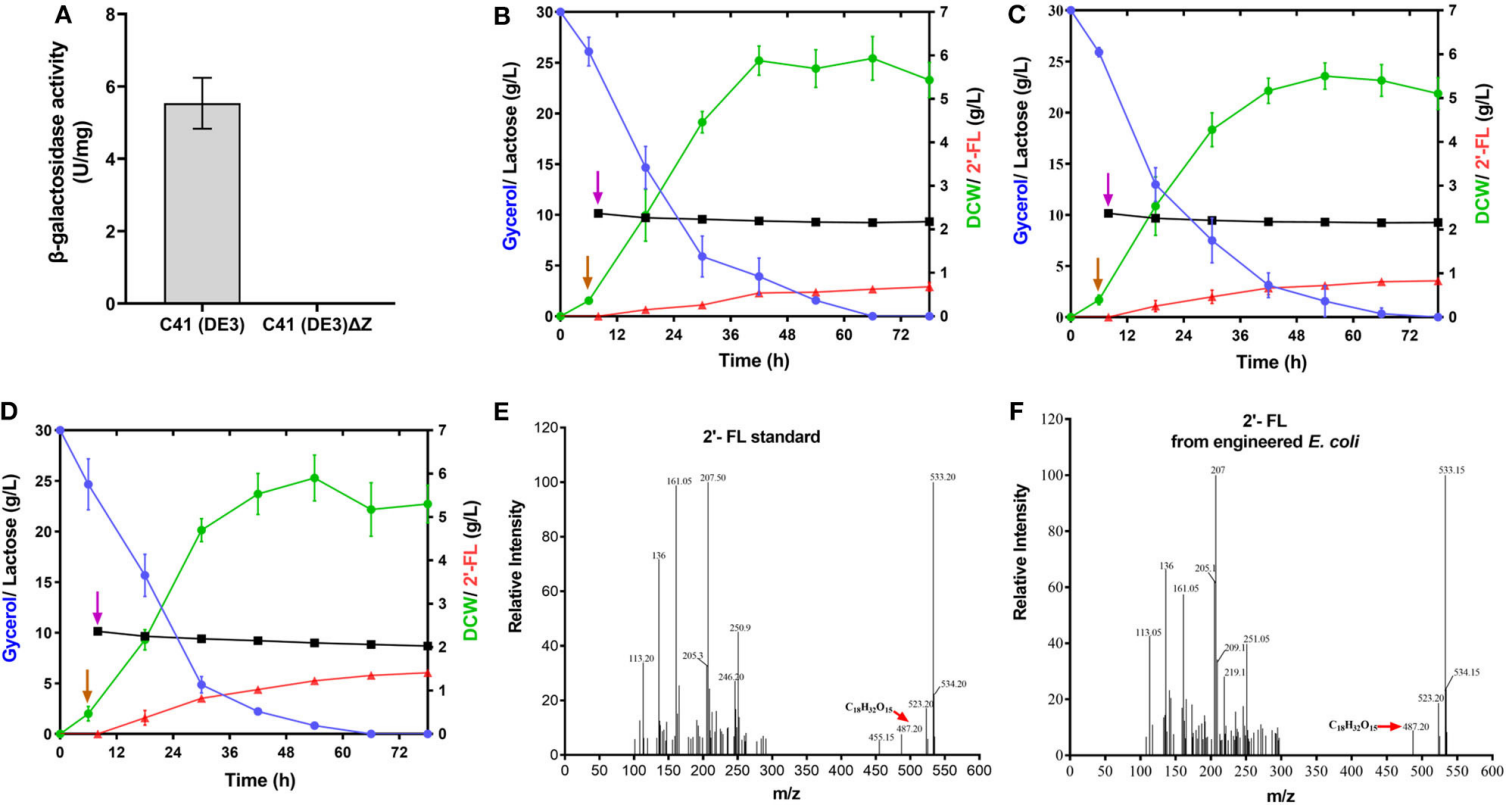
Production of 2′-FL in the recombinant *E. coli* C41 (DE3)ΔZ by fed–batch fermentation. **(A)** Specific activities of β-galactosidase from the C41 (DE3) and C41 (DE3)ΔZ strains. **(B–D)** Time profiles of recombinant strains, C41ΔZ/pA, C41ΔZ/pC, and C41ΔZ/pR during the batch fermentations. Symbols are: lactose (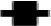), glycerol (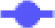), 2′-FL (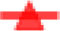), and DCW (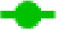). Brown vertical arrow indicates the time point for IPTG induction. Purple vertical arrow indicates the time point for lactose addition. **(E,F)** LC/MS identification of 2′-FL produced by the recombinant *E. coli* C41 (DE3)ΔZ. The red arrows represent the 2′-FL debris from the mass spectrometer.

In view of the fact that the presence of the active β-galactosidase encoded by *lacZ* in the host cells will limit the utilization of the substrate lactose (Chin et al., 2015; Huang et al., 2017), we did a detection for the β-galactosidase activity of *E. coli* C41 (DE3)ΔZ strain. As shown in Figure 2A, no β-galactosidase activity was observed in C41(DE3)ΔZ, indicating that the host strain is indeed incapable of lactose catabolism. To produce 2′-FL from lactose and glycerol via the *de novo* pathway of GDP-L-fucose biosynthesis, the genes, *manB, manC, gmd, wcaG*, and *futC*, were co-expressed in host strain through different combinations of recombinant plasmids, resulting in the engineered strains C41ΔZ/pA, C41ΔZ/pC, and C41ΔZ/pR (Table 1). Batch fermentations of these engineered strains were carried out in the 500-mL baffled shake flasks to elucidate the performance of 2′-FL synthesis. As a result, the strain C41ΔZ/pR harboring pRS-CBGW and pET-*futC* produced a highest titer of 1.41 g/L of 2′-FL with a yield of 0.67 mol 2′-FL/mole lactose within 78 h (Figure 2D and Table 2), which was prominently higher than those of the strains, C41ΔZ/pA and C41ΔZ/pC, almost 2.27-fold and 1.64-fold, respectively (Figures 2B,C and Table 2), implying a highly efficient conversion of 2′-FL from lactose. Also, these results showed that the choice of different plasmid combinations have an impact on 2′-FL synthesis. For the three plasmid candidates, pACYCDuet-1, pCDFDuet-1 and pRSFDuet-1, as well as the selected plasmid pETDuet-1, there was little difference except for the replication origins and resistance genes. Presumably, a high-copy plasmid may be more beneficial to the expression of effective proteins (Supplementary Figure 2A), thus improving GDP-L-fucose formation and 2′-FL production (Supplementary Figure 3 and Table 2). In addition, to verify synthesis of 2′-FL in the engineered strains, samples purified and enriched from the shake-flask cultures was analyzed by LC/MS. And it was clearly revealed the presence of 2′-FL produced by the above strains (Figures 2E,F). Therefore, a reliable and efficient route for 2'-FL synthesis was successfully constructed in *E. coli* C41 (DE3)ΔZ through the enhancement of endogenous GDP-L-fucose synthesis and the introduction of fucosylation activity. Considering that the combination of recombinant plasmids pRS-CBGW and pET-*futC* resulted in a higher 2′-FL yield, the resultant combination strategy was used for our subsequent work.

**Table 2:** Batch culture results obtained from the metabolically engineered *E. coli* strains in this study.

**Strains**	**Maximum DCW (g/L)**	**2′-FL concentration (g/L)**	**Lactose concentration (g/L)**	**Yield (mol 2′-FL/mol lactose)^b^**
BL21ΔlacZ/ pET-CBGF +	N.D.^c^	0.50	0.52	0.66
pCDF-*futC*^a^				
C41ΔZ/pA	5.88 ± 0.26	0.62 ± 0.101	9.33 ± 0.062	0.52 ± 0.006
C41ΔZ/pC	5.47 ± 0.14	0.86 ± 0.059	9.15 ± 0.053	0.58 ± 0.004
C41ΔZ/pR	5.53 ± 0.33	1.41 ± 0.043	8.69 ± 0.075	0.67 ± 0.002
C41ΔZW/pR	5.50 ± 0.25	2.87 ± 0.161	7.68 ± 0.122	0.79 ± 0.004
C41ΔZD/pR	5.41 ± 0.07	3.55 ± 0.052	7.17 ± 0.060	0.83 ± 0.003
C41ΔZK/pR	5.36 ± 0.32	2.64 ± 0.003	7.70 ± 0.152	0.74 ± 0.008
C41ΔZD/pRA	5.58 ± 0.13	3.91± 0.114	6.92 ± 0.034	0.83 ± 0.009
C41ΔZD/pRAB	5.72 ± 0.27	4.63 ± 0.069	6.55 ± 0.174	0.84 ± 0.004
C41ΔZD-F/pRAB	5.26 ± 0.16	5.39 ± 0.202	5.81 ± 0.133	0.88 ± 0.003

a *The result of batch fermentation of engineered E. coli BL21 (DE3) ΔlacZ expressing manB, manC, gmd, fcl, and futC reported by a previous study (Huang et al., 2017)*.

b *2′-FL yield was calculated from extracellular 2′-FL concentration determined and lactose consumption*.

c *N.D., not detect*.

### Improving Production by Modulating the Availability of the Precursor GDP-L-Fucose

**Figure 3:**
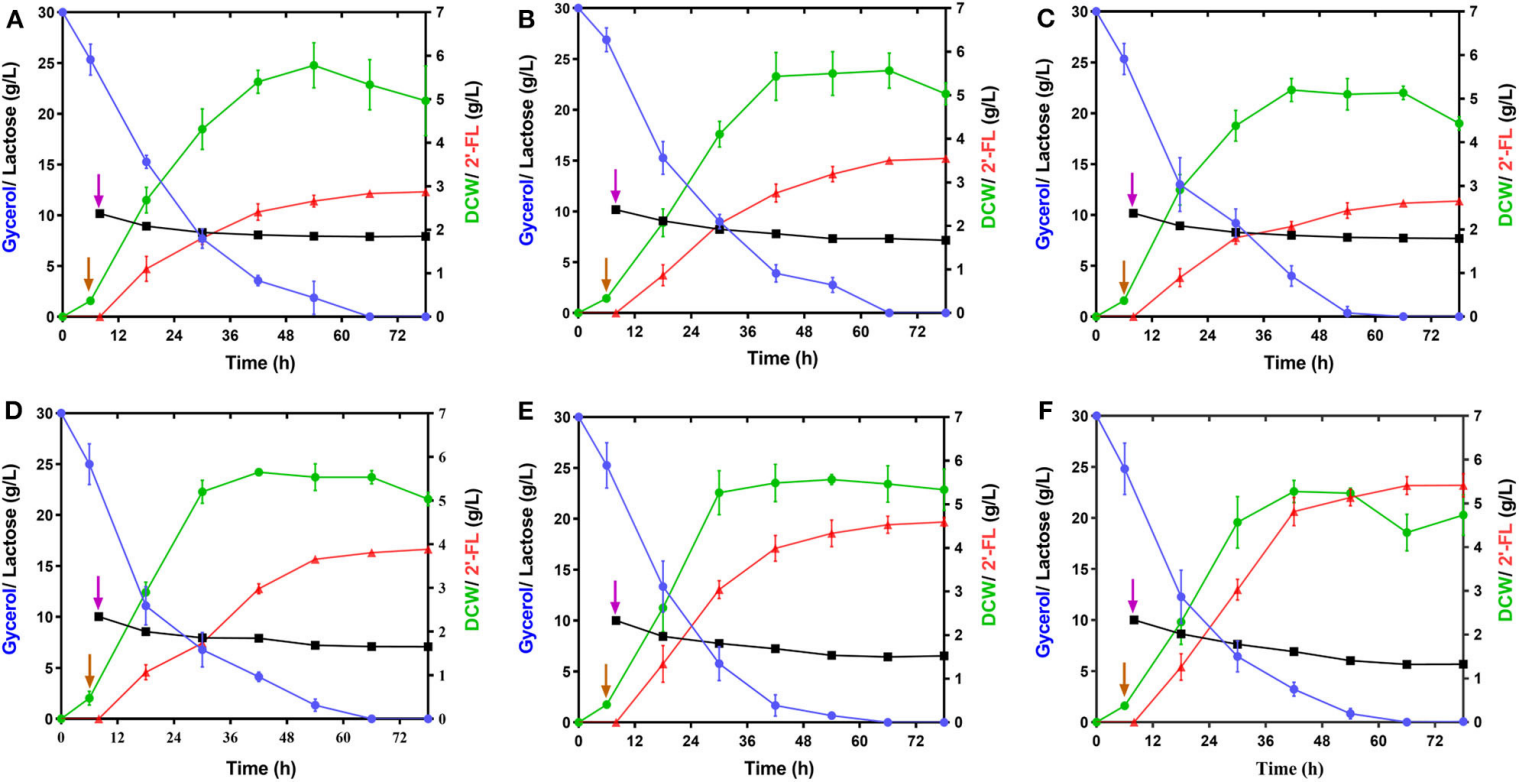
Effect of the modulations of intracellular GDP-L-fucose availability and fucosylation activity on 2′-FL production. **(A–E)** Time profiles of the engineered strains, C41ΔZW/pR, C41ΔZD/pR, C41ΔZK/pR, C41ΔZD/pRA, and C41ΔZD/pRAB during the batch fermentations. **(F)** Time profile of the strain C41ΔZD-F/pRAB harboring a genomic integration of an individual copy of *futC* during the batch fermentation. Symbols are: lactose (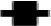), glycerol (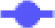), 2′-FL (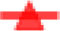), and DCW (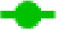). Brown vertical arrow indicates the time point for IPTG induction. Purple vertical arrow indicates the time point for lactose addition.

GDP-L-fucose, as a donor of L-fucose, has always been an essential substrate in the biosynthetic reaction of fucosylated oligosaccharides. In this study, its availability within the producer cells determines the overall yield of 2′-FL. Although this precursor can be synthesized by *E. coli* itself via the *de novo* pathway, the carbon flux along this pathway only produced trace amounts of GDP-L-fucose inside wild-type *E. coli* cells for the biosynthesis of colanic acid (Stevenson et al., 1996). Thus, to enable more GDP-L-fucose to enter 2′-FL synthesis instead of being converted into clonic acid, in the present work, the *wacJ* gene involved in colanic acid biosynthesis was knocked out by using a scarless CRISPR-Cas9 editing system, yielding the strain C41ΔZW. The *wcaJ*-deleted strain C41ΔZW/pR accumulated around 8.6 mg/L of intracellular GDP-L-fucose in the batch fermentation (Supplementary Figure 3), which was higher than the levels of several other studies (Dumon et al., 2001; Huang et al., 2017). Moreover, inactivation of this competitive pathway led to a substantial increase in the 2'-FL titer, reaching 2.87 g/L, which was about 2.04 times higher than that obtained in the control strain C41ΔZ/pR (Figure 3A and Table 2). Meanwhile, the yield of 2′-FL from lactose was also increased to 0.79 mol 2′-FL/mol lactose in the strain C41ΔZW/pR (Table 2), indicating that more GDP-L-fucose intercepted from the competitive pathway can facilitate the synthesis of 2′-FL when lactose was sufficiently supplied in our production system.

In addition, GDP-D-mannose is an important intermediate involved in GDP-L-fucose biosynthesis in the *de novo* pathway (Figure 1). It is reported that there are two additional ways to hydrolyze GDP-D-mannose in *E.coli*, i.e., GDP-mannose mannosyl hydrolase encoded by *nudD* gene and GDP-mannose hydrolase encoded by *nudk* gene, both of them can participate in the regulation of cell wall biosynthesis by converting GDP-D-mannose into different sugar molecules (McLennan, 2006; Xu et al., 2006). In this work, to direct more carbon flux from GDP-D-mannose toward GDP-L-fucose pool and its downstream 2′-FL synthesis, single gene *nudD* and *nudK* were deleted, respectively, leading to the corresponding strains C41ΔZWD and C41ΔZWK. As a consequence, the knockout of *nudD* gene generated an improved GDP-L-fucose concentration in C41ΔZWK/pR (Supplementary Figure 3), and a maximum titer of 2′-FL was appreciably increased to 3.55 g/L (Figure 3B), which was 23.7% higher than that detected in the control strain C41ΔZW/pR. Moreover, nearly 3.0 g/L lactose consumption enabled further increase in 2′-FL yield from lactose to 0.83 mol/mol (Table 2). In contast, a slightly decrease in GDP-L-fucose accumulation was observed in the *nudK-*mutant *E.coli* C41ΔZWD/pR but accompanied by 2′-FL production with a titer of 2.64 g/L in the end of fermentation (Supplementary Figure 3 and Figure 3C). This finding was unexpected with regard to the known characteristics of GDP-mannose hydrolase NudK, indicating that the *nudK* gene exerted other unexplained functions that necessarily regulate the formation of GDP-L-fucose.

Noteworthily, RcsA and RcsB both are responsible for the transcriptional regulation of colanic acid synthesis in *E. coli* (Wehland and Bernhard, 2000). Overexpression of *rcsA* could lead to an up-regulation in the expression levels of colanic acid synthesis genes (Gottesman and Stout, 1991) and increased the intracellular GDP-L-fucose (Huang et al., 2017), but unfortunately, the preferred strain *E. coli* C41 (DE3) in this study has no native *rcsA* and *rcsB* genes. For these reasons, we introduced the heterogenous *rcsA* and *rcsB* from *E. coli* MG1655 into the strain C41ΔZWD. Protein profiles showed that available RcsA could be expressed individually in the strain C41ΔZWD/pRA (Supplementary Figure 2B), or co-expressed with RcsB in the strain C41ΔZWD/pRAB (Supplementary Figure 2C). Inevitably, the intracellular GDP-L-fucose levels in both the strains were further increased (Supplementary Figure 3). Moreover, the *rcsA*-expressing strain promoted the extracellular accumulation of 3.91 g/L 2′-FL (Figure 3D), but only exhibited a slight improvement (Table 2). Interestingly, the 2′-FL concentration was dramatically elevated to 4.63 g/L in the strain co-expressing *rcsA* and *rcsB* (Figure 3E), which was 30.4% higher than that detected in the control strain (Table 2). In addition, around 3.2 and 3.6 g/L lactose were consumed by the two strains, respectively, but almost identical high yields were achieved finally (Table 2). As indicated above, the further increased carbon flux toward the GDP-L-fucose pool along the *de novo* pathway encouraged more 2′-FL synthesis.

### Integration of an Individual *futC* for Enhancing Fucosylation Activity

Although above strains gave a great progress in 2′-FL production, the intracellular GDP-L-fucose was also accumulated synchronously, suggesting that the fucosylation activity in the engineered cells may tend to be saturated, which can be explained at least partially by the fact that α*-*1,2-FT was mainly expressed as insoluble form in the *E. coli* (Supplementary Figure 2) (Lee et al., 2012a; Baumgärtner et al., 2013). Here, to balance more GDP-L-fucose into 2′-FL synthesis cells, an individual copy of *futC* gene was inserted into the *fucIK* gene locus of the engineered host, resulting in the strain C41ΔZWD*-*F. As a result, 5.39 g/L of 2′-FL was produced from about 4.3 g/L of lactose consumed by the recombinant strain C41ΔZWD*-*F/pRAB (Figure 3F), which showed a moderate increase (~16.5%) in titer compared with that obtained in the control strain. Moreover, a final yield of 2′-FL was increased to 0.88 mol/mol from lactose (Table 2), indicating that an additional α-1,2-FT activity advantageously promoted the intracellular fucosylation of more lactose.

### Strengthening the Production by Optimizing the Fermentation Conditions

**Figure 4:**
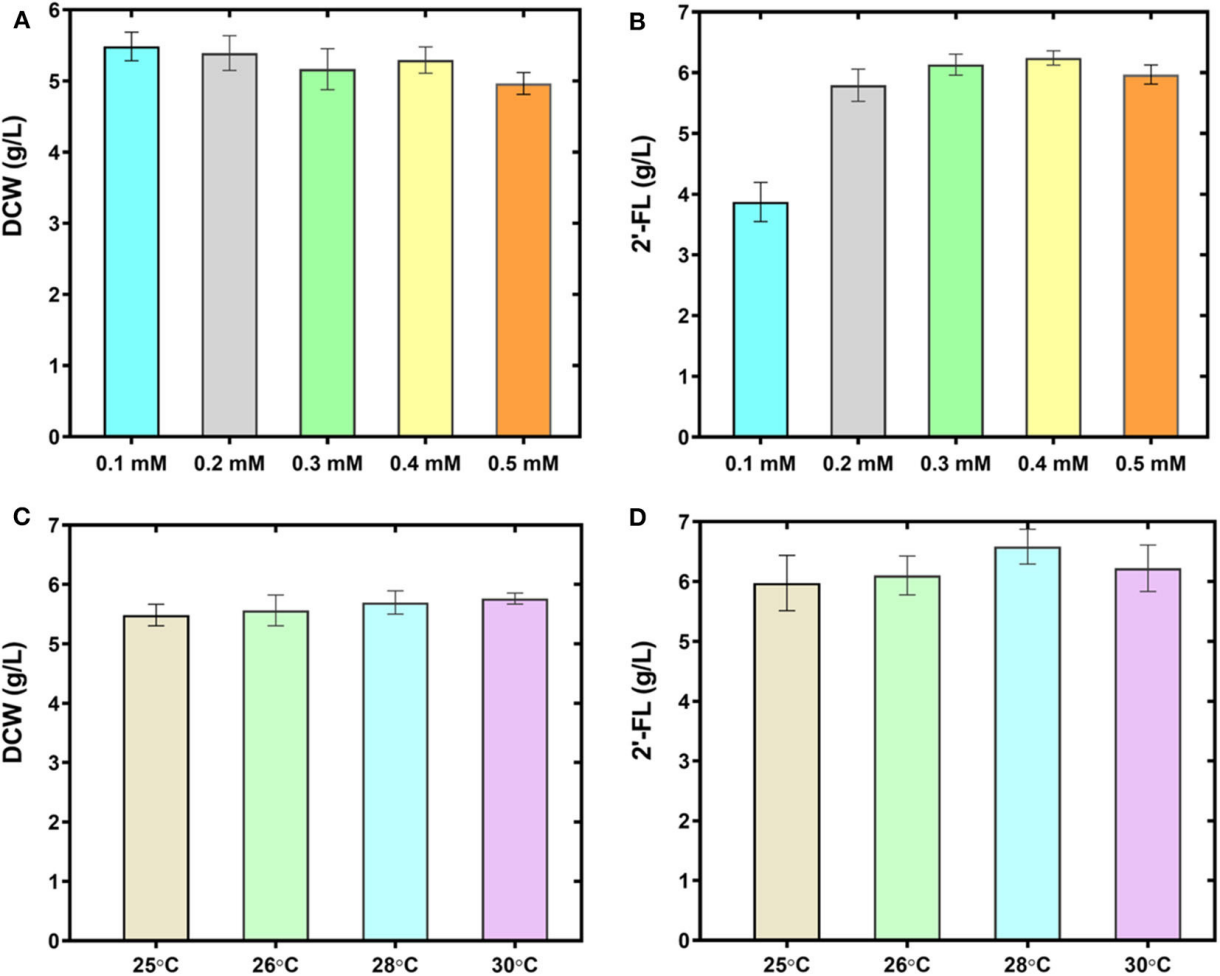
Improving 2′-FL production by optimizing the fermentation conditions. Biomass **(A)** and 2′-FL concentration **(B)** of the strain C41ΔZWD-F/pRAB at different IPTG concentrations. Biomass **(C)** and 2′-FL concentration **(D)** of the strain C41ΔZWD-F/pRAB at different culture temperatures.

The host strain C41 (DE3) derivative used in this study has a lower transcription rate of T7 RNA polymerase involved in T7 promoter-mediated gene expression than that of BL21 (DE3) strain. To give full play to the production performance of the engineered strain C41ΔZWD*-*F co-expressing the genes *manB, manC, gmd, wcaG, futC, rcsA*, and *rcsB*, various fermentation conditions, including carbon sources, IPTG concentration and incubation temperature, were optimized in the 500-mL baffled shake flasks. The effect of equal concentration of glucose instead of glycerol as the sole carbon source on 2′-FL production was explored, however, the level of 2′-FL was severely inhibited in the defined medium I (Supplementary Figure 5). Concurrently, the protein bands corresponding to the predicted sizes of interest were also not observed in SDS-PAGE analysis (Data not shown), revealing that the induced expression of key enzymes involved in the 2′-FL synthesis system was badly disturbed. These results were evidently inconsistent with those of previous studies (Lee et al., 2011, 2012b; Huang et al., 2017). In addition, the effect of various concentrations of IPTG (including 0.1, 0.2, 0.3, 0.4, and 0.5 mM) on 2′-FL production were evaluated. Eventually it seemed that addition of 0.5 mM IPTG had a weakly negative effect on cell biomass (Figure 4A), while production of 2′-FL was greatly accelerated as IPTG concentration increased from 0.1 to 0.5 mM, and a highest titer of 6.32 g/L 2′-FL was produced at 0.4 mM IPTG (Figure 4B). Furthermore, at 0.3 mM IPTG, 2′-FL titer was also as high as 6.26 g/L. Considering the cost of IPTG and its adverse effects on producer cells, 0.3 mM IPTG is preferentially employed as the optimal induction concentration. On the other hand, different culture temperatures (including 25°C, 26°C, 28°C, 30°C) were also assessed at 0.3 mM IPTG. As shown in Figures 4C,D, when the temperature was increased to 28°C, a maximum 2′-FL titer was achieved, up to 6.86 g/L, with a yield of about 0.92 mol/mol from lactose. However, a further increase in the IPTG concentration or culture temperatures did not favor product accumulation. In brief, a preliminary optimal condition was obtained with our continuous efforts, that is, the strain C41ΔZWD*-*F/pRAB enabled a best fermentation performance at 0.3 mM IPTG and 28°C in the shake flasks with glycerol as the sole carbon source.

### Production of 2′-FL by Fed-Batch Fermentation

**Figure 5:**
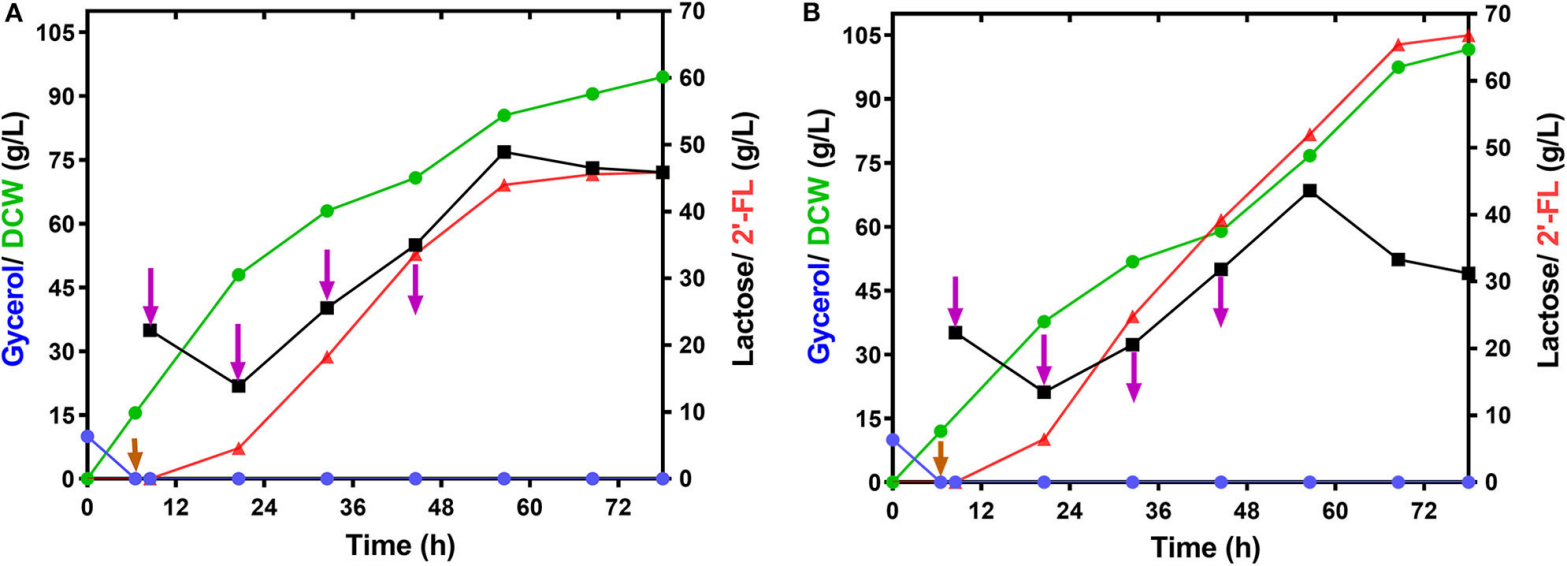
Time profiles of the fed-batch fermentation by the strain *E.coli* C41ΔZWD-F/pRAB in 50-L bioreactor. **(A)** Cultivation was carried out at 25°C and 0.2 mM IPTG Induction. **(B)** Cultivation was carried out at 28°C and 0.3 mM IPTG Induction. Symbols are: lactose (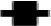), glycerol (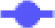), 2′-FL (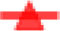), and DCW (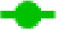). Brown vertical arrow indicates the time point for IPTG induction. Purple vertical arrow indicates the time point for lactose addition.

To demonstrate the performance of large-scale 2′-FL production with the engineered strain C41ΔZWD-F/pRAB, fed-batch fermentations were executed in a 50-L bioreactor under the optimized and non-optimized conditions, respectively. By consumption of glycerol, a final biomass reached 94.50 g/L, and a maximum 2′-FL concentration produced at 0.2 mM IPTG and 25°C was 45.58 g/L with a yield of 0.85 mol/mol and a productivity of 0.65 g/L/h (Figure 5A and Table 3). By comparison, a pretty 2′-FL production was increased linearly up to 66.80 g/L with a final DCW of 101.24 g/L at 0.3 mM IPTG and 28°C (Figure 5B). Also, a higher yield of 0.89 mol 2′-FL/mol lactose, and a higher productivity of ~0.95 g/L/h were obtained at the end of fed-batch fermentation (Table 3).

**Table 3:** Comparison of results of fed-batch fermentations of engineered *E. coli* C41ΔZWD-F/pRAB under different conditions.

**Strains**	**IPTG concentration (mM)**	**Culture temperature (°C)**	**DCW (g/L) (g/L)**	**2′-FL concentration**	**Yield (mol 2′-FL/mol lactose)^a^**	**Productivity (g/L/h)^b^**
ΔL YA BCGW-W^c^	0.1	25	57.60	15.4	0.6	0.53
C41ΔZWD-F/pRAB	0.2	25	94.50	45.58	0.85	0.65
	0.3	28	101.24	66.8	0.89	0.95

a *2′-FL yield was calculated from extracellular 2′-FL concentration determined and lactose consumption*.

b *2'-FL productivity was calculated during the 2-FL production period after lactose dumping*.

c *The previous result (Chin et al., 2017) of fed-batch fermentation of engineered E. coli BL21 (DE3)ΔlacZ Tn7:lacYA expressing manB, manC, gmd, wcaG, and wcfB (from B. fragilis) was cited*.

## Discussion

As a major structural category in HMOs family, fucosylated oligosaccharides are known to have vital protective effects infants and adults (Petschacher and Nidetzky, 2016; Sprenger et al., 2017). Typically, the most abundant 2′-FL has been widely studied and commercialized in the dairy products of certain manufacturers, such as Jennewein, Glycom A/S, Abbott, Nestlé and Wyeth Nutrition (Nestlé, 2019; Abbott Laboratories, 2020; Wyeth Nutrition, 2020). Consequently, a sufficient amount of 2′-FL must be provided at a reasonable price to meet the broader needs for basic research and practical applications. Despite previous attempts to facilitate 2′-FL production in *E. coli* (Sprenger et al., 2017; Bych et al., 2019), yet some metabolic disadvantages limit the efficient synthesis of 2′-FL. To develop an efficient and stable microbial process of 2′-FL production on an industrial scale, in this study, we proposed a reliable 2′-FL-producing cell factory in the *E. coli* C41 (DE3) derivatives by manipulating several key synthetic pathways.

Above all, lactose is a commonly available substrate, and readily assimilated into *E. coli* cells for manufacturing 2′-FL. Previous studies observed that the strains incapable of utilizing lactose, or with weakened lactose utilization, could produce increased amounts of 2′-FL (Chin et al., 2015, 2017). In the case of C41(DE3) ΔZ strain, the loss of lactose hydrolysis activity allowed a higher 2′-FL production than *lacZ*-mutant *E. coli* BL21star(DE3) (Table 2; Huang et al., 2017), probably because of the differences between production systems, including expression plasmids, production hosts, culture conditions, etc. In addition, an attempt was also made to overexpress the lactose permease gene, *lacY*, to boost the lactose uptake. Strangely, a transient increase in intracellular lactose concentration in a short period was observed (Supplementary Figure 4), but the 2′-FL production and biomass in the lacY-overexpressed strain was decreased significantly (Data not shown), which is inconsistent with others' observation (Huang et al., 2017), and may be attributed to a cytotoxicity induced by excessive lactose uptake (Dykhuizen and Hartl, 1978; Patel et al., 1982).

GDP-L-fucose is a necessary precursor for the biosynthesis of fucosylated oligosaccharides, and its intracellular availability is of great importance for the efficient production of 2′-FL. It has been mentioned that direct addition of GDP-L-fucose *in vitro* into the fermentation system can markedly increase the production of 2′-FL (Huang et al., 2017). However, owing to its scarcity and high price, this feeding strategy is not economically viable for directing the cellular metabolism toward the desired 2′-FL production. In this study, we screened a pair of efficient plasmid combination, pRS-CBGW and pET-*futC*, that overexpressing GDP-L-fucose *de novo* synthesis gene and α-1,2-FT gene, resulting in higher levels of GDP-L-fucose and 2′-FL (Supplementary Figure 3 and Table 2). Subsequently, the knockout of *wcaJ* gene involved in the colanic acid biosynthesis remarkably alleviated an undesirable loss of GDP-L-fucose, resulting in a great improvement in 2′-FL production (Figure 3B and Table 2). Subsequently, for the first time we inactivated two different GDP-mannose hydrolysis pathways encoded by *nudD* and *nudK* gene, respectively. As expected, deletion of *nudD* gene blocked the conversion of GDP-L-mannose to D-mannose, and enabled more carbon flux from GDP-D-mannose toward GDP-L-fucose pool, which resulted in a remarkable increase in the intracellular GDP-L-fucose and its downstream 2′-FL levels (Supplementary Figure 3 and Table 2). Unexpectedly, however, the absence of *nudK* gene failed to improve GDP-L-fucose and 2′-FL production in our study (Supplementary Figure 3 and Table 2). Since the biological roles of this hydrolase are not yet fully clear, further research should be considered in our subsequent work. In addition, we introduced the co-expression of *rcsA* and *rcsB* for synergistically up-regulating the GDP-L-fucose biosynthesis. The introduction of RcsA alone merely produced a marginal increase (~10.4 %) in 2′-FL yield (Table 2), which is lower than that (58.2%) reported by Huang et al. (2017), whereas the co-expression of *rcsA* and *rcsB* encouraged a greater increase in 2′-FL production (Table 2), which might be ascribed to the fact that the interaction of RcsA with RcsB is supposed to enhance the stability and activity of RcsA (Ebel and Trempy, 1999; Wehland and Bernhard, 2000). Besides, it is said that in *E. coli*, RcsB is absolutely required to regulate glutamate-dependent acid resistance (GDAR), and this acid tolerance mechanism may contribute to cell growth and target product synthesis, especially in shake flask experiments (Castanié-Cornet et al., 2010).

Fucosyltransferases are involved in the final step of biosynthesis of fucosylated oligosaccharides by transferring L-fucose from GDP-L-fucose to the acceptor substrates. As we've seen, the production performance of 2′-FL was further improved with the integration of an individual *futC* expression cassette into the chromosome (Figure 3F and Table 2), however, no observable reduction in intracellular GDP-L-fucose level was detected (Supplementary Figure 3), which may be due to the alleviation of the feedback inhibition of GDP-L-fucose to the Gmd enzyme through the increased fucosylation reaction (Somoza et al., 2000).

Generally, differences in fermentation conditions have a conspicuous impact on the evaluation of the production performance of engineered bacteria. In the present work, we acquired a set of optimal fermentation process by simply optimizing different fermentation parameters, including carbon sources, inducer concentration, and culture temperature. In terms of using glucose instead of glycerol as the sole carbon source for 2′-FL production, obviously it is impracticable in our production system (Supplementary Figure 5). This phenomenon can be explained by the repression of carbon metabolism caused by the glucose effect (Ma et al., 2010), thus interfering with the lactose transport and protein expression for 2′-FL biosynthesis. Regarding IPTG concentration and culture temperature, undoubtedly, both of them directly or indirectly affect the expression of recombinant protein and cell viability. It turned out that a high amount of IPTG may impose a metabolic burden on the cells and even reduce their growth (Kosinski et al., 1992). Similarly, it is believed that strong carbon fluxes triggered by an excessive temperature may cause the production of certain undesirable metabolites, which adversely affects cell growth, protein and target product production (Vemuri et al., 2006; Chong et al., 2013). Actually, our investigation avoided the “high” values of concentration and temperature (Figure 4), so that the above conclusions cannot be drawn from our data. Nevertheless, we obtained a practically best level for 2′-FL production, where an excellent production performance was achieved in a 50-L bioreactor through fed-batch fermentation with our optimized conditions in 78 h (Figure 5B and Table 3), which is quite superior to the results reported by Chin et al. (2016, 2017). This sharp increase breaks through the current level of coverage reported in a laboratory scale. In addition, it is noteworthy that the biomass increased continuously during the whole fermentation process, and no growth inhibition or cell lysis was observed like that of BL21 (Figures 5A,B), indicating an expected stability of our production system. Still, more efforts will be needed to reproduce the production performance in large-scale fermentation systems.

## Conclusion

In this study, we successfully developed an engineered cell factory for efficient 2′-FL production using inexpensive glycerol as a carbon feedstock and lactose as a precursor by collectively taking into account the intracellular lactose utilization, GDP-L-fucose availability, and fucosylation activity. Finally, the resultant strain C41ΔZWD-F/pRAB surprisingly produced a maximum titer of 66.80 g/L 2′-FL with a yield of 0.89 mol/mol from lactose in 50-L fed-batch fermentation bioreactor, which was nearly 9.74-fold that produced in the batch fermentation. To our best knowledge, this is the highest level of 2′-FL production reported on the laboratory scale to date. This production performance richly presents the feasibility of our efforts, which will provide a favorable candidate process for further industrial production. In addition, more efforts should be considered in the future, such as genetic stability of engineered strains, the regeneration of key cofactors GTP and NADPH, and subsequent thorough optimization of fermentation process, to achieve a more economical and even better yield of 2′-FL.

## Data Availability Statement

The raw data supporting the conclusions of this article will be made available by the authors, without undue reservation.

## Author Contributions

ZN and JY conceived and designed the research. ZN wrote the manuscript. ZN, ZL, YL, and YG performed the experiments and analyzed the data. JW contributed the host strain *E. coli* C41 (DE3)ΔZ. LY contributed analytical tools. XC and JY supervised the project and critically revised the manuscript. All authors read and approved the manuscript.

## Conflict of Interest

JW, LY, and XC were employed by the company Wuhan Zhongke Optics Valley Green Biotechnology Co. Ltd. The remaining authors declare that the research was conducted in the absence of any commercial or financial relationships that could be construed as a potential conflict of interest.
