# Supplementary material for: Multi-Path Optimization for Efficient Production of 2′-Fucosyllactose in an Engineered Escherichia coli C41 (DE3) Derivative
Source: Front Bioeng Biotechnol. 2020 Dec 3;8:611900. doi: 10.3389/fbioe.2020.611900 (PMC7793955; doi:10.3389/fbioe.2020.611900)
Supplement: Supplementary file 1 [file Data_Sheet_1.docx]

Supplementary Material

# Supplementary Tables and Figures

## Supplementary Tables

**Supplementary Table 1 |** Oligonucleotides and DNA fragments used in this study.

| **Primers** | | **Sequences(5' to 3')** |
| --- | --- | --- |
| **Primers for gene cloning** | | |
| CB-F | gtataagaaggagatatacatATGGCGCAGTCGAAACTCTATC | |
| CB-R | ggtggcagcagcctaggTTACTCGTTCAGCAACGTCAG | |
| CB-T-F | ctttaataaggagatataccATGGCGCAGTCGAAACTCTATC | |
| CB-T-R | cgacttaagcattatgcggccGCATGCCTGAGGTTTCAGC | |
| GW-F | gtataagaaggagatatacatATGTCAAAAGTCGCTCTCATC | |
| GW-R | ggtggcagcagcctaggTTACCCCCGAAAGCGGTCTTG | |
| FT-F | gtataagaaggagatatacatATGGATGATGATGCATTCAAAG | |
| FT-R | ggtggcagcagcctaggTTAGGCGTTATATTTCTGAC | |
| FT-FKF-F | ggaactaaggaggatattcaTTGACAATTAATCATCCGGCTCG | |
| FT-FKF-R | cttcatttttaatttttgcggccgCCGAAAGAGTTTGTAGAAACGC | |
| FT-fucIK-F | tccgccttagcagagcgccaataaagcgagatgaggaatcctgatGTGTAGGCTGGAGCTGCTTC | |
| FT-fucIK-R | gcatattggctttaatctgattccacaatgtgttgcgacttcctcCGAAAGAGTTTGTAGAAACGC | |
| *rcsA*-F | ctttaagaaggagatataccATGTCAACGATTATTATGGAT | |
| *rcsA*-R | gcattatgcggccgcaagcttAGCGCATGTTGACAAAAATACC | |
| *rcsB*-F | gtcaacatgcgctaagcttgcATGAACAATATGAACGTAATTATTGC | |
| *rcsB*-R | gacttaagcattatgcggccTTAGTCTTTATCTGCCGGAC | |
| **Primers for the construction of specific pTargetF** | | |
| *wcaj*-gRNA-F | **GCTGGTGGTGTTCCAGATGT**GTTTTAGAGCTAGAAATAGC | |
| *wcaj*-gRNA-R | **ACATCTGGAACACCACCAGC**ACTAGTATTATACCTAGGAC | |
| *nudK*-gRNA-F | **GCTGCTGGATAACGACGAAC**GTTTTAGAGCTAGAAATAGC | |
| *nudK-*gRNA-R | **GTTCGTCGTTATCCAGCAGC**ACTAGTATTATACCTAGGAC | |
| *nudD*-gRNA-F | **ACCACTCACTATGTGGTGCT**GTTTTAGAGCTAGAAATAGC | |
| *nudD*-gRNA-R | **AGCACCACATAGTGAGTGGT**ACTAGTATTATACCTAGGAC | |
| **Primers for the amplication of homologous arms of the target gene, upstream (US) and downstream (DS)** | | |
| *wcaj*-US-F | CATCGTTAATCTCTATGGTG | |
| *wcaj*-US-R | TACTCAAGGTCGAACTCGACGTCGACCAGTTGTTGCAGATTG | |
| *wcaj-*DS-F | CAATCTGCAACAACTGGTCGACGTCGAGTTCGACCTTGAGTA | |
| *wcaj-*DS-R | CAGCACGTCACCAATGAGATC | |
| *nudK-*US-F | CATGCCAGGCTCGAAGAAAG | |
| *nudK-*US-R | CAATATCTTCATCTTCGACAGGTGATTTGTTGCGTCATAC | |
| *nudK-*DS-F | GTATGACGCAACAAATCACCTGTCGAAGATGAAGATATTG | |
| *nudK*-DS-R | CAGCCGTGGCGCTATCTCCC | |
| *nudD*-US-F | CATGTGATCCCAGCATTGCT | |
| *nudD*-US-R | GTACTCCGGCACGCTTCTCATTTGCCAAGCAGAAACTCGC | |
| *nudD-*DS-F | GCGAGTTTCTGCTTGGCAAATGAGAAGCGTGCCGGAGTAC | |
| *nudD-*DS-R | GACGTTATCGACGTTATGCAG | |
| **Primers for identifying mutations in genomic DNA** | | |
| *wcaj-*IF | ACTCTGCTGACGTTGCTGAA | |
| *wcaj-*IR | CGAACTGGTGGTTGTCGATA | |
| *nudK-*IF | CGGATGTGGAAAGCCAGTAC | |
| *nudK-*IR | ATCGGATTTGTCGGATTGAG | |
| *nudD-*IF | CGTCAGGAAGACTTTGCCAC | |
| *nudD-*IR | CGTGGAGACGTTATCGACGT | |
| *fucIK*-IF | ACTGACCATCACGCATTACAGT | |
| *fucIK*-IR | GATTCCACAATGTGTTGCGAC | |
| **dsDNA fragments for the genome editing templates** | | |
| *wcaJ-*T | CATCGTTAATCTCTATGGTGCAACGCTTTTCAGATATCACCATCATGTTTGCCGGACTATGGCTGGTTTGCGAAGTCAGCGGACTGTCATTCCTCTACATGCACCTGTTGGTGGCGCTGATTACGCTGGTGGTGTTCCAGATGTTGGGCGGCATCACCGATTTTTATCGCTCATGGCGCGGTGTTCGGGCAGCGACAGAATTTGCCCTGCTGCTACAAAACTGGACCTTAAGCGTGATTTTCAGCGCCGGACTGGTGGCGTTCAACAATGATTTCGACACGCAACTGAAAATCTGGCTGGCGTGGTATGGGCTGACCAGTATCGGACTGGTGGTTTGCCGTTCATGTATTCGCATTGGGGCGGGCTGGCTGCGTAATCATGGCTATAACAAGCGCATGGTCGCCGTAGCGGGGGATTTAGCCGCCGGACAAATGCTGATGGAGAGCTTCCGTAATCAGCCGTGGTTAGGGTTTGAAGTGGTGGGCGTATACCACGACCCAAAACCGGGCGGCGTTTCTAACGACTGGGCGGGCAATCTGCAACAACTGGTCGACGTCGAGTTCGACCTTGAGTACATCCGCGAATGGAGCGTCTGGTTCGATATCAAAATCGTTTTCCTGACGGTATTCAAGGGCTTCGTTAACAAAGCGGCATATTGATATGAGCTTACGTGAAAAAACCATCAGCGGCGCGAAGTGGTCGGCGATTGCCACGGTGATCATCATCGGCCTCGGGCTGGTGCAGATGACCGTGCTGGCGCGGATTATCGACAACCACCAGTTCGGCCTGCTTACCGTGTCGCTGGTGATTATCGCGCTGGCAGATACGCTTTCTGACTTCGGTATCGCTAACTCGATTATTCAGCGAAAAGAAATCAGTCACCTGGAACTTACCACGTTGTACTGGCTGAACGTCGGGCTGGGGCTTGTGGTGTGCGTGGCGGTGTTTTTGTTGAGTGATCTCATTGGTGACGTGCTG | |
| *nudD*-T | CATGTGATCCCAGCATTGCTGCGCCGCTTCCACGAGGCGACGGCACAGAATGCACCGGACGTGGTGGTATGGGGCAGCGGTACACCGATGCGTGAATTCCTGCACGTCGATGATATGGCGGCGGCGAGCATTCATGTCATGGAGCTGGCGCATGAAGTCTGGCTGGAGAACACCCAGCCGATGCTGTCGCACATTAACGTCGGCACGGGCGTTGACTGCACCATCCGTGAACTGGCGCAAACCATCGCCAAAGTGGTGGGTTACAAAGGTCGGGTGGTTTTTGATGCCAGCAAACCGGATGGTACGCCGCGCAAACTGCTGGATGTGACGCGCCTGCATCAGCTTGGCTGGTATCACGAAATCTCACTGGAAGCGGGGCTTGCCAGCACTTACCAGTGGTTCCTTGAGAATCAAGACCGCTTTCGGGGGTAATGATGTTTTTACGTCAGGAAGACTTTGCCACGGTAGTGCGCTCCACTCCGCTTGTCTCTCTCGACTTTATTGTCGAGAACAGTCGCGGCGAGTTTCTGCTTGGCAAATGAGAAGCGTGCCGGAGTACCCGGATTATGAAAATTCTGGTCTACGGCATTAACTACTCGCCGGAGTTAACCGGCATCGGCAAATACACCGGCGAGATGGTGGAATGGCTGGCGGCACAAGGTCATGAGGTGCGGGTTATTACCGCACCGCCTTACTACCCGCAGTGGCAGGTGGGCGAGAACTATTCCGCCTGGCGCTACAAACGAGAAGAGGGGGCCGCCACGGTGTGGCGCTGCCCGCTGTACGTGCCAAAACAGCCGAGCACCCTGAAACGCTTGTTGCATCTCGGCAGTTTTGCCGTCAGCAGTTTCTTTCCACTGATGGCGCAACGTCGCTGGAAGCCGGATCGCATTATCGGCGTAGTGCCAACGCTGTTTTGCACGCCGGGAATGCGCCTGCTGGCGAAGCTCTCTGGTGCGCGTACCGTGCTGCATATTCAGGATTACGAAGTGGACGCCATGCTGGGGCTGGGCCTTGCCGGAAAAGGCAAAGGCGGCAAAGTGGCACAGCTGGCGACGGCGTTCGAACGTAGCGGACTGCATAACGTCGATAACGTC | |
| *nudK*-T | CATGCCAGGCTCGAAGAAAGAAGTGAAGAACGCCCGCGAAGAGGGGGCCAACTTCGAATTTAACGTCCAGCCGGTGGCGCTTGAGCTGAATGAACAAGGTCACGTCTGCGGGATTCGTTTCCTGCGCACGCGTCTTGGAGAGCCGGATGCCCAGGGGCGTCGGCGTCCAGTGCCGGTGGAAGGCAGTGAATTTGTCATGCCAGCCGACGCGGTGATTATGGCGTTTGGCTTCAATCCGCACGGGATGCCGTGGCTGGAGTCGCACGGTGTAACGGTAGACAAATGGGGCCGCATCATCGCGGATGTGGAAAGCCAGTACCGTTACCAGACCACCAATCCGAAAATCTTCGCTGGTGGTGACGCCGTGCGTGGTGCGGATCTGGTGGTTACCGCAATGGCAGAAGGACGTCATGCGGCACAGGGGATTATTGACTGGCTGGGGGTAAAATCAGTCAAATCTCACTGATAGCCTGCGCAGACAAACCCGACTTCACAGCGTAAGATAATTGTTCATTTCGCGCTGTGGAGTCGGTATGACGCAACAAATCACCTGTCGAAGATGAAGATATTGAAGTGCTCGAGCTGCCGTTCAGCCAGGCGCTGGAGATGATCAAAACCGGCGAGATACGTGACGGTAAGACGGTGTTATTGCTTAACTATTTGCAAACGTCACATTTAATGGACTGAAAAATAACAATAATATTTCGTTGTTTATTATTGGCTCAATCCGACAAATCCGATTGAGCCGCGCTACCTGCGCAACGAAGATACGACTTGTGCTGTTTGTTTGAACTTCTGGGGTCGTACCGTCCATGCGCTATCGCATTTTCCTTCTCTTTTTTTTCGCTTTGTTGCCGACGTCTTTGGTGTGGGCGGCACCAGCGCAACGGGCGTTTTCCGACTGGCAGGTCACCTGTAATAACCAAAATTTCTGCGTGGCGCGTAATACGGGCGATCATAATGGACTGGTGATGACCCTGAGCCGCAGCGCCGGGGCGCATACCGATGCCGTTTTACGTATTGAGCGCGGCGGATTGAAGTCGCCGGAGGCGTCAGAAGGGGAGATAGCGCCACGGCTG | |

Lowercase letters represent the short homologous sequences for DNA recombination; Bold letters indicate a 20bp spacer sequence specific for each target gene.

**Supplementary Table 2 |**Summary of 2′-FL production by metabolically engineered microorganisms.

| **Hosts** | **Engineering strategies** | **Titer** | **References** |
| --- | --- | --- | --- |
| *E. coli* JM109(DE3) | Overexpressing the genes for endogenous GDP-L-fucose biosynthetic enzymes and heterologous fucosyltransferase (FucT2) | 1.23 g/l | Lee et al., 2012a |
| *E. coli* JM109 | Integrating the genes responsible for GDP-L-fucose de novo synthesis, two copies of *futC* gene*, and fkp* gene into the chromosome of *E. coli* JM109 | 20.28 ± 0.83 g/l | Baumgärtner et al., 2013 |
| *E. coli* BL21star(DE3) | Deleting the whole endogenous lactose operon and introducing the modified lactose operon containing lacZΔM15 from *E.coli* K-12; overexpressing the genes for GDP-L-fucose biosynthetic enzymes and FucT2 with three aspartate molecules at the N-terminal | 6.4 g/L | Chin et al., 2015 |
| *E. coli* BL21star(DE3) | Deleting *lacZ* and *fucI-fucK* genes,and expressing *fkp* and *futC* genes | 23.1 g/L | Chin et al., 2016 |
| *E. coli* BL21star(DE3) | Deleting *lacZ* and overexpressing the genes for endogenous GDP-L-fucose biosynthetic enzymes and heterologous fucosyltransferase (WcfB) | 15.4 g/L | Chin et al., 2017 |
| *E. coli* BL21star(DE3) | Overexpressing the genes for endogenous GDP-L-fucose biosynthetic enzymes and FucT2; deleting *lacZ, lon,* and *wcaJ* genes; overexpressing *lacY* and *rcsA* genes; engineering the cofactor NADPH regeneration pathway | 9.12 g/L | Huang et al., 2017 |
| *Saccharomyces cerevisiae* | Introducing three heterologous genes (*fkp, fucT2,*and *LAC12* coding for lactose permease) | 0.50 g/L | Yu et al., 2018 |
| *Saccharomyces cerevisiae* | Introducing the heterologous genes (*gmd, wcaG, fucT2,*and *LAC12* coding for lactose permease) | 0.51 g/L | Liu et al., 2019 |
| *Saccharomyces cerevisiae; Yarrowia lipolytica* | Installing a lactose transporter (Lac12) and enzymes (GMD/GMER) that convert GDP-mannose to GDP-fucose; Introducing the SUMOstar ® tagged variant of FutC and a transporter (CDT2) capable of exporting 2'-FL from yeast | 24.0 g/L | Hollands et al., 2019 |
| *Bacillus subtilis* | Introducing the salvage pathway gene *fkp*; overexpressing the sugar transporter gene (*glcP*) and *futC*; introducing the heterologous lactose permease to improve lactose import and deleting *yesZ* to block the degradation of lactose; engineering the cofactor GTP regeneration pathway | 5.01 g/L | Deng et al., 2019 |
| *E. coli* BL21star(DE3) | Deleting *lacZ* and *fucI-fucK* genes,and expressing *fkp* and *fucT2*; deleting of the genes (*araA* and *rhaA*) to avoid fucose isomerization | 47.0 g/L | Jung et al., 2019 |

## Supplementary Figures


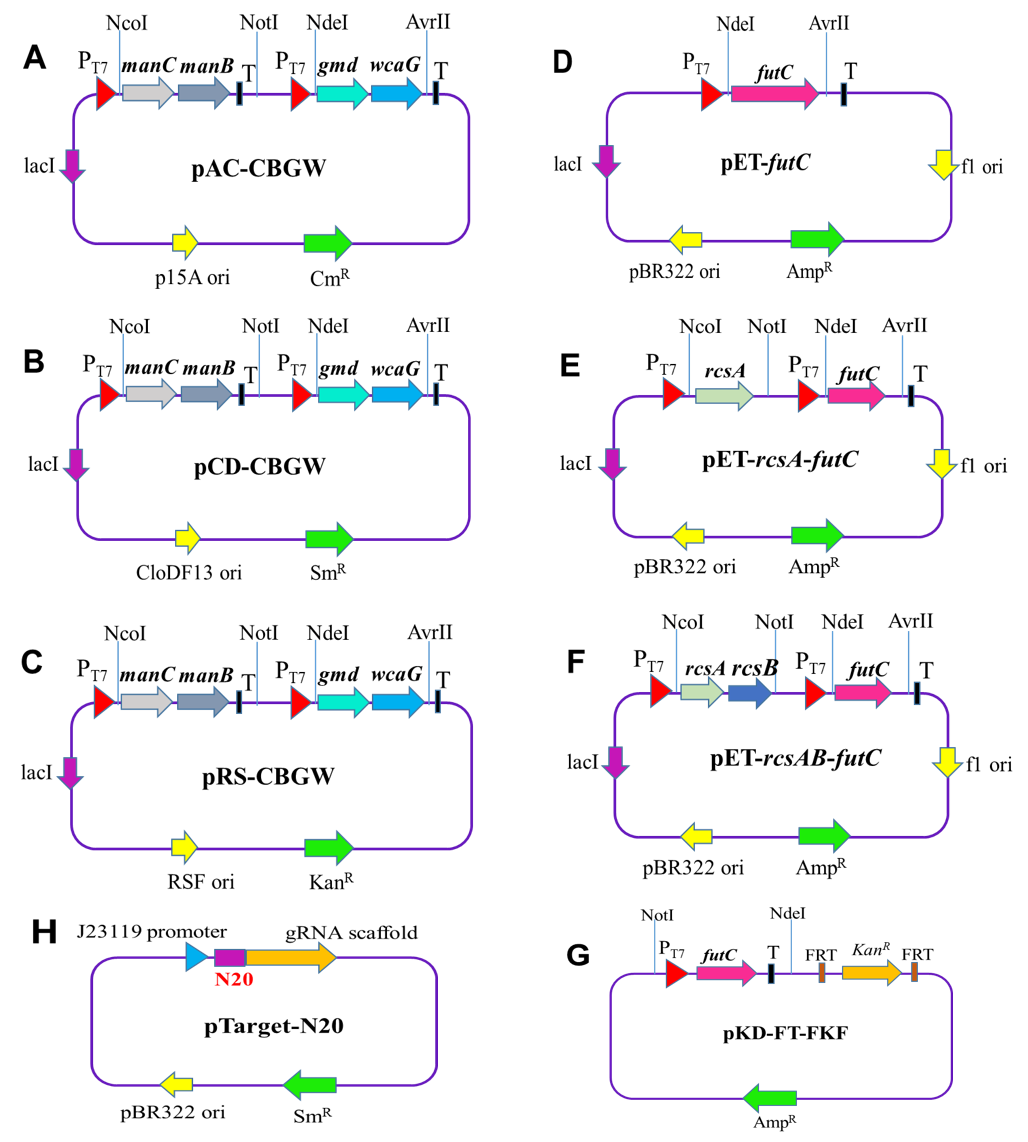


**Supplementary Figure 1 |** Recombinant plasmids constructed in this study. **(A)**-**(F)** Plasmids for overexpressing the target genes for 2*′*-FL biosynthetic pathway. **(G)** The plasmid was constructed to provide a complete *futC* gene expression cassette for its genome integration. **(H)** pTargetF plasmid series used in single-gene modification with 20 bp target sequence of gene loci of interest. N20, 20 bp target sequence specific for each target gene (including *wcaJ, nudK, nudD*), can be complementary to the targeting region; gRNA scaffold, guide RNA scaffold for directing Cas9 protein to the targeted region.

**
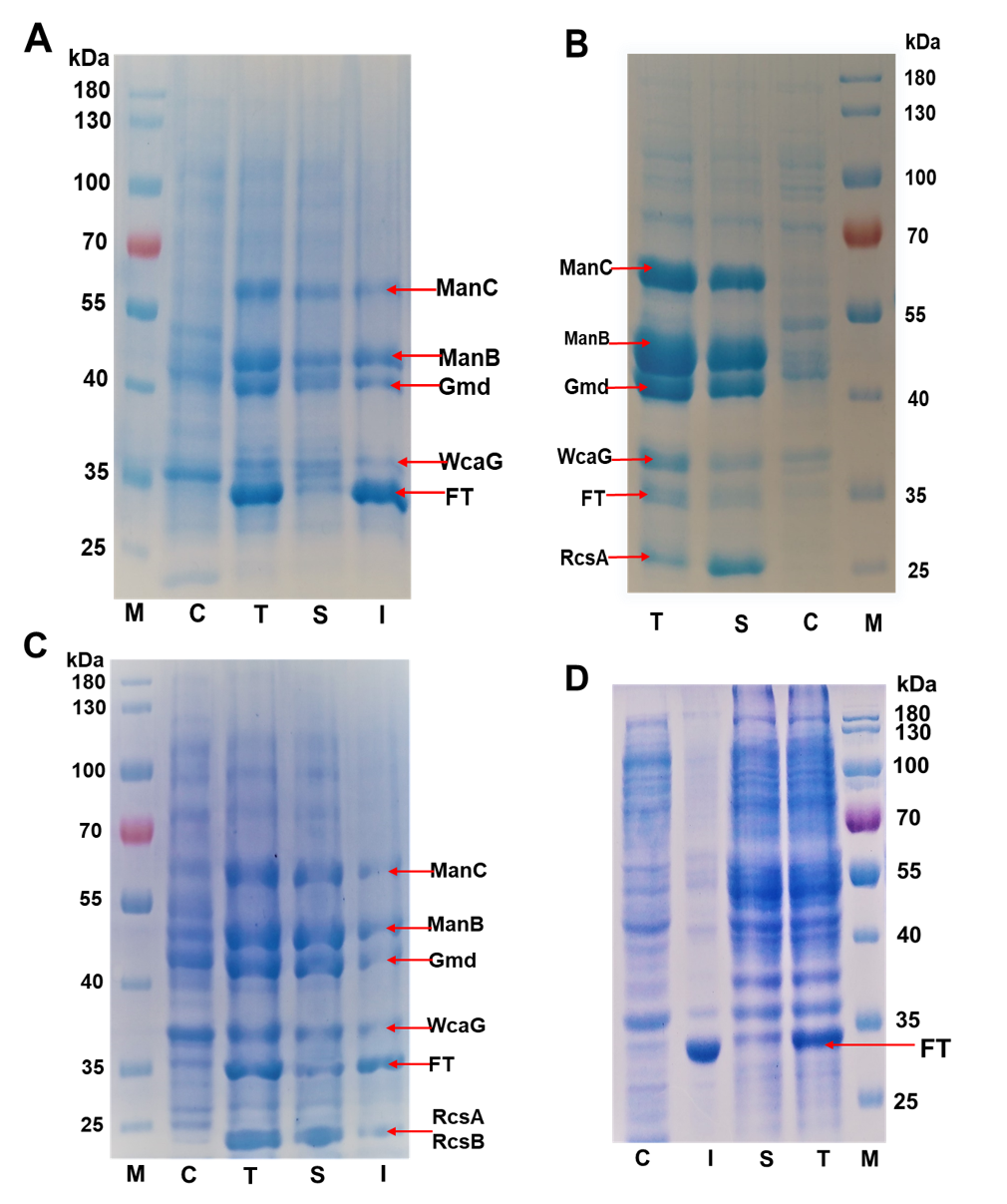
**

**Supplementary Figure 2 |** SDS-PAGE analysis of disrupted cell extracts from the engineered *E. coli* strains C41ΔZ/pR **(A)**, C41ΔZWD/pRA **(B)**, C41ΔZWD/pRAB **(C)**, and C41ΔZWD-F **(D)**, respectively. Cells were harvested after 8 h of 0.4 mM IPTG induction. The abbreviations were defined as follows: **C**, a control sample from the corresponding strain without IPTG induction; **T,** total protein fraction; **S,** soluble protein fraction; **I**, insoluble protein fraction; **M**, molecular weight marker. The red arrows beside the protein names indicate the corresponding protein bands with the estimated sizes.

**
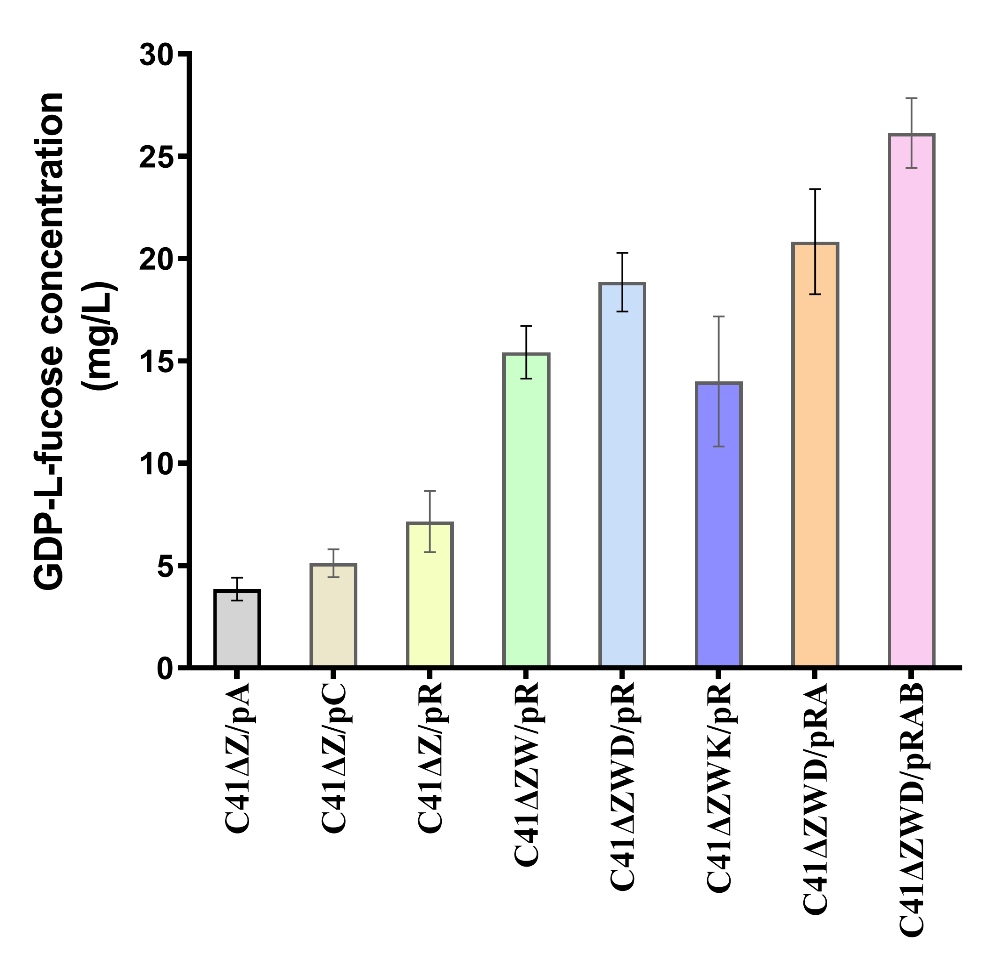
**

**Supplementary Figure 3 |** Intracellular concentrations of GDP-L-fucose from the engineered strains after 24 h induction by the batch fermentation.

**
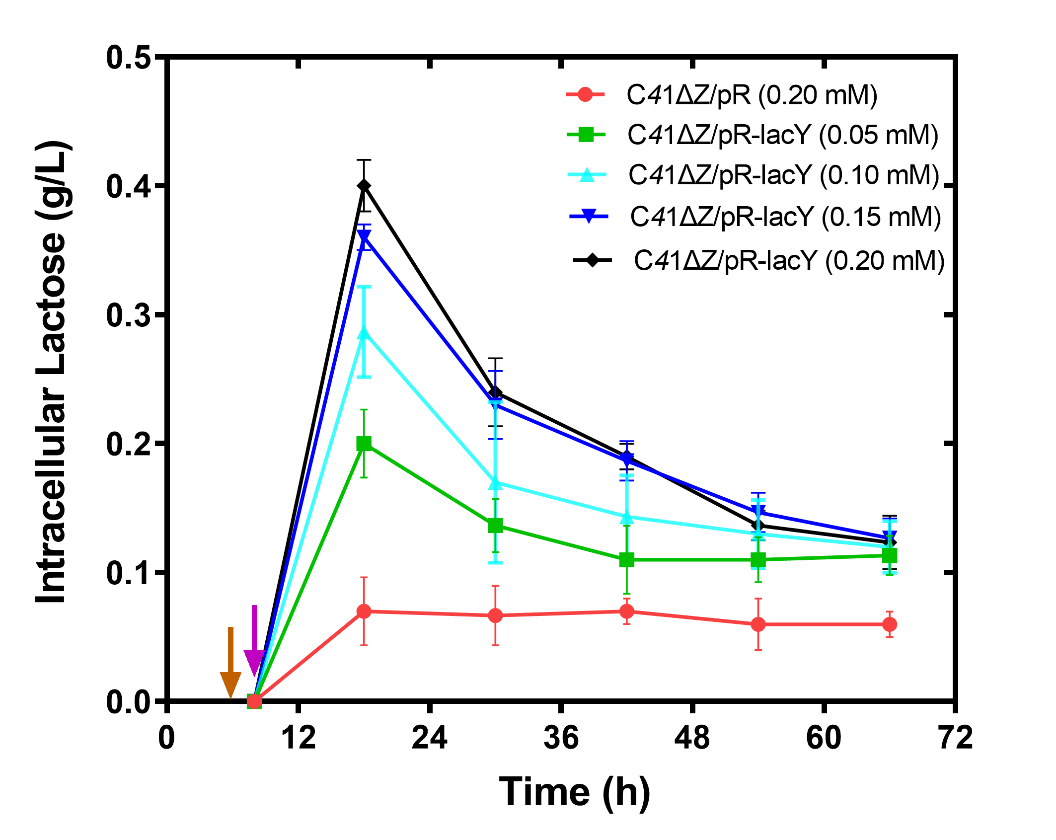
**

**Supplementary Figure 4 |** Intracellular lactose concentration in C41ΔZ/pR (control strain) and C41ΔZ/pR-lacY (*lacY*-overexpressed strain). Batch fermentation was regularly performed in the 500-mL baffled shake flasks. When OD600 reached ~0.8, IPTG was added to at different final concentrations, including 0.05 mM, 0.10 mM, 0.15 mM, 0.20 mM (brown vertical arrow), and then 10 g/L lactose was injected after an additional 2 h of incubation (purple vertical arrow). Subsequently, samples were taken once for each 12h incubation.

**
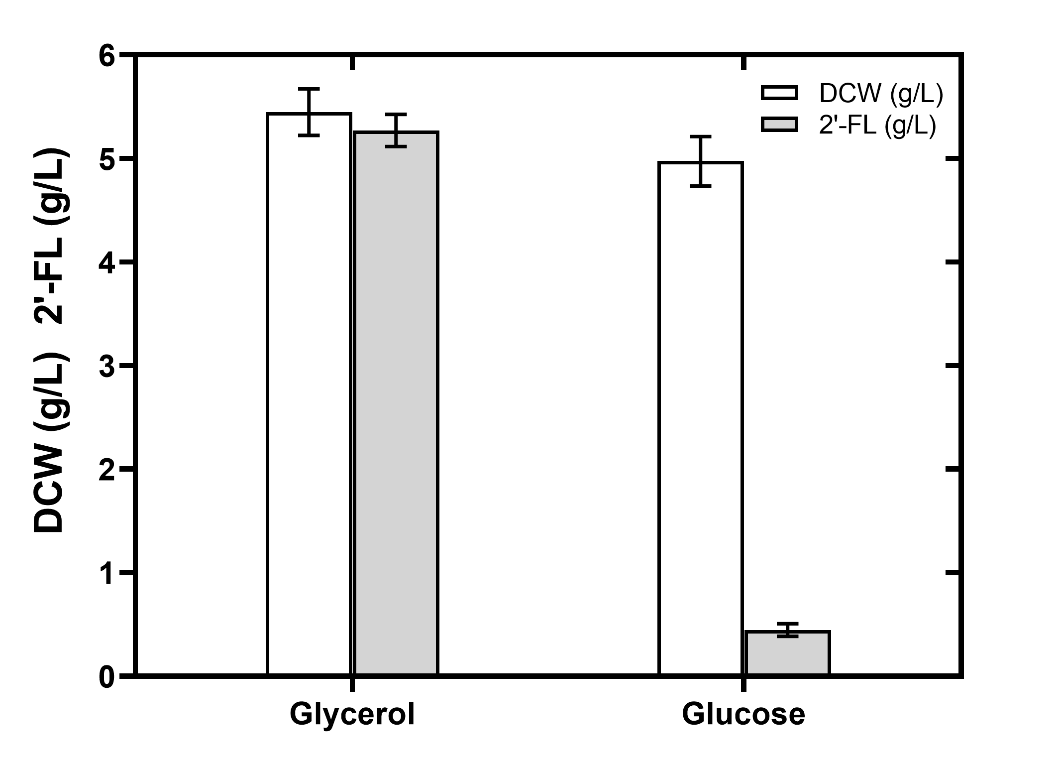
**

**Supplementary Figure 5 |** Comparison of the results of batch fermentation (78 h) with the strain C41∆ZWD-F/pRAB in different carbon sources.

# Supplementary References

Hollands, K., Baron, C. M., Gibson, K. J., Kelly, K. J., Krasley, E. A., Laffend, L. A., Lauchli, R. M., Maggio-Hall, L. A., Nelson, M. J., Prasad, J. C., Ren, Y., Rice, B. A., Rice, G. H., & Rothman, S. C. (2019). Engineering two species of yeast as cell factories for 2'-fucosyllactose. *Metab. Eng*. 52, 232–242. [doi: org/10.1016/j.ymben.2018.12.005](https://doi.org/10.1016/j.ymben.2018.12.005).

Liu, J. J., Kwak, S., Pathanibul, P., Lee, J. W., Yu, S., Yun, E. J., Lim, H., Kim, K. H., & Jin, Y. S. (2018). Biosynthesis of a Functional Human Milk Oligosaccharide, 2'-Fucosyllactose, and l-Fucose Using Engineered *Saccharomyces cerevisiae.* *ACS Synth. Biol.* 7, 2529–2536. [doi: org/10.1021/acssynbio.8b00134](https://doi.org/10.1021/acssynbio.8b00134).
